# Asymptomatic Empty Sella: A Literature Review and Suggestions for Evaluation in Clinical Practice

**DOI:** 10.7759/cureus.75965

**Published:** 2024-12-18

**Authors:** Tyler E Rice-Canetto, Patrick Carroll, Louis Reier, Javed Siddiqi

**Affiliations:** 1 Neurosurgery, Arrowhead Regional Medical Center, Colton, USA; 2 Neurosurgery, California University of Science and Medicine, Colton, USA; 3 Neurosurgery, Riverside University Health System Medical Center, Moreno Valley, USA; 4 Neurosurgery, Desert Regional Medical Center, Palm Springs, USA

**Keywords:** arachnoidocele, empty sella, empty sella turcica, endocrine manifestations, neurological manifestations, ophthalmological manifestations, pituitary gland abnormalities, primary empty sella syndrome, secondary empty sella syndrome, sella turcica

## Abstract

Empty sella (ES) is a radiographic finding defined by the presence of cerebrospinal fluid in the sella turcica, with associated compression of the pituitary gland. Empty sella syndrome (ESS) is the combination of this radiographic finding with endocrine, ophthalmological, and/or neurological symptoms. The focus of this literature review is to synthesize information about asymptomatic or incidental ES specifically, meaning the radiologic finding of an empty sella without symptoms. This condition is typically discovered incidentally through imaging when patients present with unrelated pathologies. Our intention is to strengthen the existing body of work on this clinical presentation and call for a standardized management protocol of ES. This literature review was performed in concordance with the Preferred Reporting Items for Systematic Review and Meta-Analyses (PRISMA). Data were extracted on paper characteristics, epidemiology, diagnostics, management, and outcomes.

Overall, patients with radiographic findings of ES have a good prognosis. The majority of patients never become symptomatic (progression to ESS), and if symptoms do arise, they usually have little impact on quality of life. However, despite its inherent asymptomatic nature, given ES's estimated prevalence within society, the possibility of a critical underlying pathology, and the potential for subsequent development of consequential symptoms, this paper calls for the implementation of a standardized management protocol. We suggest that all patients with this finding and an unidentified underlying etiology, at a minimum, receive an endocrine panel for evaluation of pituitary function and a referral to ophthalmology for formal assessment of papilledema. We then recommend tailored management and referrals based on the results of these evaluations, as well as treatment of any subsequent symptomatology. While these are our recommendations based on a thorough review of the literature, further studies are necessary to reach a consensus on best clinical practices.

## Introduction and background

The term Empty sella (ES) was first used by Busch in 1951, in which vertical enlargement of the sella turcica was noted in 40 of 788 human cadavers with no known pituitary disease [1]. Kaufman presented a number of cases and proposed that ES was caused by fluctuations in intracranial pressure (ICP) in the intrasellar subarachnoid space, leading to bony remodeling [2]. Sage et al. examined 156 sphenoid bones from those with known ES and histologically normal pituitary glands, noting an incomplete diaphragma sellae in more than 50% of cases [3]. In recent years, increases in the quality and quantity of radiologic studies have expanded the identification of asymptomatic (or incidental) ES [4]. 

ES is characterized by flattening of the pituitary gland and enlargement of the sella turcica due to excess cerebrospinal fluid (CSF) [5,6]. ES may be subdivided into various categories, including primary empty sella (PES) vs secondary empty sella (SES), as well as complete vs partial ES. PES is postulated to be from a congenital defect in the diaphragma sellae with associated elevations in ICP, whereas SES is secondary to direct damage to the pituitary gland [7]. The difference between partial and complete ES is purely radiographic. The distinction between PES and SES, as well as between complete and partial ES, is further discussed in the classifications section of this paper. The terms 'empty sella' and 'empty sella syndrome' (ESS) are frequently used interchangeably; however, the meanings are different and should be clearly defined. ES refers solely to a radiographic finding, whereas ESS is when the radiographic finding of ES is accompanied by endocrine, ophthalmological, and/or neurological symptoms [7].

With better technology and imaging modalities, there has been a rise in the prevalence of ES seen on radiographs. However, there is limited research to help guide practitioners with management. The goal of this paper is to provide a review of ES and provide the average practitioner with a management algorithm to follow when this radiographic finding is present.

## Review

Methods 

Data Sources and Search Strategies

To complete our literature review on ES, we conducted a comprehensive search of relevant articles in the PubMed database. We conducted a simple search on PubMed, and our search terms included the following: 'Incidental Empty Sella Syndrome,' 'Asymptomatic Empty Sella Syndrome,' 'Incidental Empty Sella,' 'Asymptomatic Empty Sella,' 'Incidental Arachnoidocele,' and 'Asymptomatic Arachnoidocele. No search filters other than the date were applied. Because “Empty Sella” and “arachnoidocele” are synonymous, we included both terms to broaden the results of our search. The use of “syndrome” implies an accompanying constellation of neurological, ophthalmological, and/or endocrine symptoms, so we excluded this term from some of our additional search queries to ensure that we were capturing our topic of interest. 

Inclusion and Exclusion Criteria

Our review included publications that specifically addressed “asymptomatic” or “incidental” ES. The aim of our search was to incorporate articles that were focused on presentations of ES without endocrine, ophthalmological, and/or neurological manifestations. Such cases were typically discovered through imaging to investigate a different clinical presentation and suspected etiology. Amongst these articles, our primary focus was papers that emphasized diagnostics and management of this condition. We included papers meeting these criteria, which were published in English and spanned a variety of publication types, including case reports, literature reviews, systematic reviews, and meta-analyses. We did not include articles that did not meet the aforementioned inclusion criteria, in addition to those meeting one or more of the following exclusion criteria: papers limited to a pediatric population (<18 years) or a geriatric population (>65 years), animal and laboratory studies, clinical trials focused on novel treatments of ESS without mention of asymptomatic ES, papers published in non-peer-reviewed journals such as editorials and opinion columns, papers requiring translation for inclusion, or papers requiring payment for inclusion.

Data Extraction

Each publication was independently screened by two reviewers to determine eligibility based on the inclusion and exclusion criteria. Articles were first screened by title and abstract, followed by a separate screening process of the complete publication content. The screened papers were split between the reviewers, and data was extracted on the following variables of interest: publication year, study design, age, sex, prevalence, patient-reported symptoms if present, diagnostic method, diagnostic findings, treatment, and outcomes.

Search of the Literature

**Figure 1:**
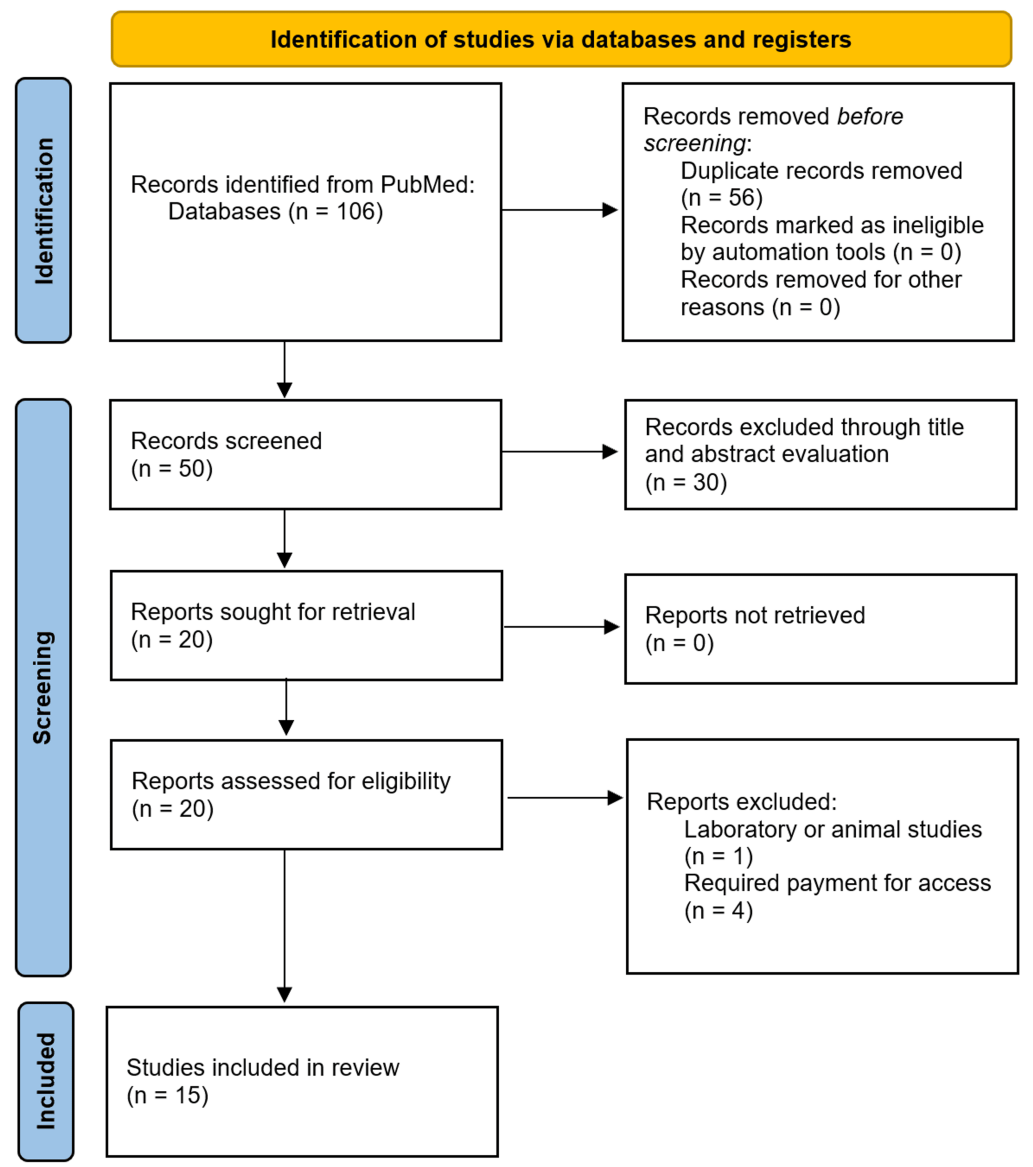
PRISMA diagram. PRISMA: (Preferred Reporting Items for Systematic Reviews and Meta-Analyses).

Our search of PubMed was conducted on April 16th, 2024. Our search terms yielded a total of 106 publications. The number of results returned by each of the search phases was as follows. The search term “Incidental Empty Sella Syndrome” yielded 21 results, “Asymptomatic Empty Sella Syndrome” returned 20 results, “Incidental Empty Sella” resulted in 32 papers, “Asymptomatic Empty Sella” yielded 29 results, “Incidental Arachnoidocele” returned two results, and lastly “Asymptomatic Arachnoidocele” resulted in an additional two papers. When we examined these initial 106 papers and eliminated any duplicate publications (56 in total), we were left with a total of 50 papers. Through our initial screening process of article titles and abstracts, we eliminated any papers that did not have our topic of interest as their main focus, and we were left with 20 articles. Upon a more in-depth review of the remaining publications, an additional five articles were excluded from our review. This screening process resulted in our final number of 15 papers, from which our authors then extracted variables of interest. Figure 1 reveals our PRISMA diagram, which further details our publication selection process. Table 1 includes details of each paper included in our review.

**Table 1:** Final papers included in review based on PRISMA diagram. PMID: PubMed identifier, ES: empty sella, NA: not applicable, CT: computed tomography, MRI: magnetic resonance imaging, ICP: intracranial pressure, BMI: body mass index, IH: intracranial hypertension, TBI: traumatic brain injury, TIA: transient ischemic attack, ICT: intracranial tension, SNHL: sensorineural hearing loss, post-op: postoperative, PES: primary empty sella, EKG: electrocardiogram.

Author and publication year	Article title	PMID	Study design	Age	Sex	Prevalence of ES in the study population or general population	Prevalence of specified findings	Clinical presentation	Patient-reported symptoms	Diagnostic method	Treatment	Outcome
Constantinescu et al., 2021 [4]	Pituitary incidentaloma	34687911	Literature review	NA	NA	NA	10% of patients harbor a pituitary incidentaloma	NA	NA	CT head, MRI brain	NA	NA
Miljic et al., 2000 [5]	Empty sella	30321014	Book chapter	"Middle-aged"	5:1 female: male ratio	79% of patients were diagnosed incidentally	NA	Obesity, hypertension	Headache, visual disturbances	CT head, MRI brain	Referral to endocrinology, osmotic diuretics or acetazolamide for patients with elevated ICP, weight loss for patients with obesity or sleep apnea, neurosurgical corrections for patients with rhinorrhea and causes of empty sella that increase ICP or cause visual disturbances, hormone replacement	NA
Ucciferro et al., 2024 [6]	Empty sella syndrome	31082046	Book	40-60 years range	5:1 female: male ratio	NA	40% of patients had hormonal abnormalities, 90% of patients had intracranial hypertension	Asymptomatic, hypertension, hormonal abnormalities including gynecomastia/acromegaly/hypogonadism, papilledema, obesity-associated symptoms including increased BMI/sleep apnea	None, headache, visual disturbances including diplopia/decreased acuity, dizziness, syncopal episodes, amenorrhea, galactorrhea, erectile dysfunction, polydipsia, polyuria, cognitive impairment	CT head, MRI brain	Supportive care and hormonal corrections for symptomatic patients, follow-up every 2-3 years for asymptomatic patients	Prognosis is dependent on hormonal imbalances, little clinical progression if asymptomatic, typically does not affect life expectancy.
Lundholm, 2024 [7]	A comprehensive review of empty sella and empty sella syndrome	38484938	Literature review	NA	NA	Incidental radiographic findings of an empty sella are prevalent in up to 35% of the general population.	NA	A subset of patients exhibits endocrine or neuro-ophthalmologic manifestation.s	NA	CT head, MRI brain	Isolated empty sella finding does not require treatment; empty sella syndrome may require pharmacologic or surgical interventions by a multidisciplinary team of neurologists, neurosurgeons, and ophthalmologists	Isolated empty sella does not benefit from treatment and is unlikely to ever require treatment
Auer et al., 2018 [8]	Primary empty sella syndrome and the prevalence of hormonal dysregulation	29510819	Systematic review	48±12 years average for study #1, 50.1±9.3 years average for study #2, 48±1 years average for study #3, 49.9±14.5 years average for study #4	"Female predominance"	6-20% estimated prevalence in the overall population	52% of patients had hypopitutiarianism	NA	Headaches, visual disturbances, rhinorrhea, sexual dysfunction, fatigue, myalgia, arthralgia, nausea, weight loss, amenorrhea	CT head, MRI brain	NA	NA
Chen et al., 2021 [9]	Prevalence of incidentally detected signs of intracranial hypertension on magnetic resonance imaging and their association with papilledema	33871552	Cross-sectional study	49.5 years median	63.5% female, 36.5% male	33.1% prevalence in the study population	NA	Surveillance of brain neoplasm, non-headache neurological symptoms, multiple sclerosis, new or worsening headache, known disorder of raised intracranial pressure	41% reported a history of headache	MRI brain, fundus photography	NA	Magnetic resonance imaging signs of IH were common among patients undergoing brain MRI in this study but rarely associated with papilledema.
Carosi et al., 2022 [10]	A multicenter cohort study in patients with primary empty sella: hormonal and neuroradiological features over a long follow-up	35813618	Retrospective cohort	51.5±16 years average, 13-84 years range	63% female, 27% male	6-20% of cases were discovered by autopsy, 8-35% estimated prevalence in the general population	40.5% of patients had hypopituitarism, 29% of patients had hormonal abnormalities	Asymptomatic, neurologic symptoms, ophthalmological symptoms, otolaryngological symptoms	Neurological symptoms, visual disturbances, various endocrine disorders	CT head, MRI brain	Hormonal evaluation and corrections, re-evaluate patients 24-36 months post-diagnosis	3% of patients developed one or more new pituitary symptoms over 6 months, 8.4% of patients showed recovery of one or more pituitary symptoms, 6.1% had a progression from partial empty sella to total empty sella
Ekhzaimy et al., 2020 [11]	Clinical presentation, evaluation and case management of primary empty sella syndrome: a retrospective analysis of 10-year single-center patient data	32943019	Retrospective cohort	46.7±17.3 years average for males, 48.8±14.1 years average for females	3.8:1, female: male ratio	79% of cases diagnosed incidentally, 42% of incidental cases were symptomatic	NA	Headache, TBI, stroke, TIA	Headache, visual disturbances, menstrual abnormalities, tinnitus, nausea, growth delay, dizziness, vertigo, seizures, neurological symptoms, acute behavioral changes, delusions, memory loss, myalgia	MRI brain	Referral to endocrinology, hormonal work-up	NA
Debnath et al., 2016 [12]	Empty sella on routine MRI studies: an incidental finding or otherwise?	26900220	Retrospective cohort	23 patients <30 years, 110 patients 30-50 years, 108 patients >50 years	139 males, 102 females	1.94% prevalence in the study population	NA	Patients undergoing MRI brain	Hormonal disturbances, headache, sensorineural hearing loss, seizures, vertigo, psychiatric disorders, visual disturbances, ataxia and raised intracranial tension	MRI brain	NA	Hormonal disturbances, psychiatric disorders, raised ICT and SNHL have been found to be more often associated with ES as compared to general population
Delegado-Hernandez et al., 2015 [13]	Analysis of the joint and a posteriori probability between primary empty sella, its comorbidities, and audiovestibular pathology	26194748	Retrospective cohort	61.78±10.77 years average	88% female, 12% male	9% prevalence in the study population	NA	Vertigo, dizziness	Vertigo, dizziness	MRI brain, audiovestublar testing	NA	NA
Ajayan et al., 2021 [14]	An incidental empty sella causing acute posterior pituitary hypofunction in a patient presenting for neurosurgery	33642303	Case report and literature review	54 years	Female	NA	25-30% of literature review patients had hypopituitarism; the case report patient did not	Asymptomatic, neurological symptoms, papilledema	Left-sided hearing loss, tinnitus, chronic intermittent headache with associated nausea	CT head, MRI brain	Craniotomy and decompression for the brain mass without any steroids prior	Diabetes insipidus for three days post-op treated with vasopressin; at three months follow-up post-op, no features of hormonal deficiencies
Pansare et al., 2021 [15]	PES syndrome presenting as severe hyponatremia in an asymptomatic septuagenarian	33505736	Case report	71 years	Male	NA	NA	A male with feminine features and short stature, referred for hyponatremia	None	MRI brain	Levothyroxine and hydrocortisone	Improvement of hyponatremia 3 days post-treatment; patient continues to be healthy
Lah et al., 2022 [16]	Case report: primary empty Sella causing secondary adrenal insufficiency and severe yet asymptomatic hyponatremia	35957779	Case report	61 years	Male	NA	NA	Superficial venous thrombosis, hyponatremia refractory to diuresis	Right leg pain and swelling	CT abdomen, MRI brain	Cortisol replacement	Resolved hyponatremia
Braiteh et al., 2019 [17]	Sinus Bradycardia as a rare and unusual presentation of partial empty sella syndrome: a case report	31327866	Case report	66 years	Male	NA	NA	Postoperative bradycardia and neurological symptoms after spinal surgery	Dizziness, lightheadedness, cognitive slowing, fatigue, reduced libido	MRI brain, EKG, labs	Neurosurgical procedure, sent to endocrinology outpatient	NA
Al Mohareb et al., 2018 [18]	Resistance to thyroid hormone-beta co-existing with partially empty sella in a Jordanian male	30530874	Case report	34 years	Male	NA	NA	Asymptomatic	Intermittent palpitations	MRI brain, EKG, labs	Bisoprolol	The patient still complained of palpitations after the treatment

Epidemiology

Historically, ES was considered a rare finding, but with advances in neuroimaging, it is now estimated to be present in 2-35% of the general population [2,7,8]. The incidence is highest in the 4th to 6th decade and is more prevalent in women by over a 4:1 ratio [19].

The incidence and prevalence of hypopituitarism in the general population are not precisely known but were evaluated amongst 146,000 adults in northwestern Spain between 1992 and 1999 [20]. Incidence was reported as 4.21 cases per 100,000 person-years, and prevalence was reported as 45.5 cases per 100,000 persons [10]. Auer et al. carried out a systematic review to estimate the prevalence of hormonal disturbances in patients with PES, finding a relative frequency of 52% [8]. There is an unexplained discrepancy between the low prevalence of hypopituitarism in the general population and the reported high prevalence of hypopituitarism amongst patients with PES. These studies show that many people who appear to be asymptomatic in the setting of radiographic ES may actually have hypopituitarism if tested. Thus, if pituitary labs were routinely drawn on everyone with ES, a proportion of those people who were thought to be asymptomatic with an incidental finding of ES would actually have ESS. However, pituitary labs aren’t routinely obtained in all patients with ES, meaning this diagnosis gets missed. 

Anatomy and physiology of the sella turcica and pituitary gland

The sella turcica, also known as the pituitary fossa, is a concave midline indentation on the superior surface of the sphenoid bone in which the pituitary gland resides. The sella turcica is bounded superiorly by the diaphragma sellae, a double-layered sheet of dura mater with a central aperture that separates the superior aspect of the adenohypophysis from the optic chiasma. The pituitary gland, also known as the hypophysis cerebri or the master gland, is a neuroendocrine gland responsible for a wide range of endocrine functions. It is split into two distinct regions, anterior and posterior. The anterior pituitary, or adenohypophysis, consists of six cell lines: somatotrophs (release growth hormone), lactotrophs (release prolactin), corticotrophs (release adrenocorticotropic hormone), thyrotrophs (release thyroid-stimulating hormone), gonadotrophs (release luteinizing hormone and follicle-stimulating hormone), and folliculostellate cells (these do not release hormones). The posterior pituitary, or neurohypophysis, stores and releases oxytocin and antidiuretic hormone [21]. 

Typically, when hypopituitarism ensues, the first hormones to become deficient are follicle-stimulating hormone, luteinizing hormone, and growth hormone. This is because these hormones are less essential for survival compared to the other pituitary hormones and, therefore, are the first to go. Intuitively, the thyroid-stimulating hormone is the next to go, and the adrenocorticotropic hormone is the last to be impacted as its role is absolutely necessary for survival [7]. 

Classifications of empty sella

ES may be subdivided into various categories, including primary versus secondary ES and complete versus partial ES.

Primary vs Secondary Empty Sella

Primary Empty Sella (PES) is defined as ES independent of an identifiable cause [7]. The pathophysiology of PES involves intrasellar herniation of CSF through the diaphragma sellae, leading to a flattened pituitary and bony remodeling [22]. The diaphragma sellae is a double-layered sheet of dura mater that forms the roof of the sella turcica [23]. While the pathophysiology of PES is known, the etiology is not. However, evidence reveals associations with conditions such as elevated ICP, obesity, arterial hypertension, pregnancy-related alterations in pituitary gland volume, and congenital defects in the diaphragma sellae [5,7,24]. Factors that may contribute to elevated ICP include but are not limited to hydrocephalus, brain hemorrhage, space-occupying lesions, idiopathic intracranial hypertension (IIH), and numerous vasculitic conditions. IIH, also referred to as pseudotumor cerebri, is the most common associated elevated ICP pathology, with a prevalence in the general population estimated at 1 in 100,000 persons [5]. While PES is often associated with these causes of elevated ICP, it is not universally observed in those with elevated ICP, indicating a multifactorial etiology [24]. With regards to pregnancy, cyclic pituitary hyperplasia with subsequent involution may be associated with enlargement of the sella and thus the development of PES [16,25]. An incomplete diaphragma sellae secondary to a congenital defect is postulated to help facilitate the passage of CSF into the sella turcica with resultant pituitary compression [24]. These numerous associations with PES support the theory of a multifactorial etiology of the condition [14]. While ES may be associated with these various disease states, in the absence of neurological, endocrine, or ophthalmological symptoms, PES is regarded as an incidental finding and a normal variant [5]. 

Secondary empty sella (SES) is defined as ES with an identifiable insult to the pituitary gland itself, resulting in its subsequent volume reduction relative to the sella turcica and the increased presence of CSF. Imaging can thus distinguish between PES and SES by the presence of normal enhancement of the pituitary gland and stalk in the former versus visible scarring and distortion in the latter [5]. The direct damage to the pituitary seen in SES may be a consequence of mechanisms such as pituitary hemorrhage, cranial surgery (pituitary mass resection, for example), radiation therapy, pituitary apoplexy, infection of the central nervous system, congenital pituitary hypoplasia, or head trauma [7,9,26,27]. Similar to PES, SES, in the absence of endocrine, ophthalmological, and/or neurological symptoms, is still considered an incidental finding [7]. 

Partial vs Complete Empty Sella

**Figure 2:**
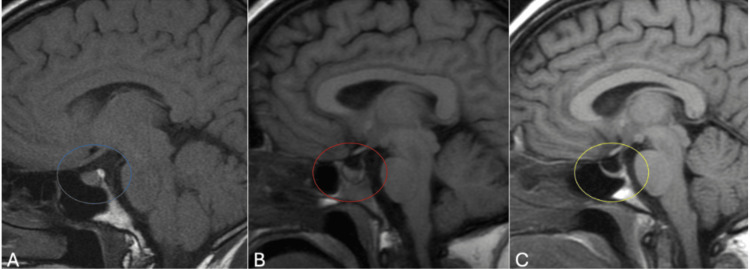
Partial vs complete empty sella on MRI. A. normal sella turcica (blue circle); B. partially empty sella turcica (red circle); C. complete empty sella turcica (yellow circle) (Taken from a free source published under Creative Commons License [28]). MRI: magnetic resonance imaging.

In addition to being subdivided into categories of PES and SES, ES can also be categorized as either complete or partial [7]. “Partial ES” means that less than 50% of the sella turcica is occupied by CSF, and the pituitary gland is at least 3 mm thick. “Complete ES” means that greater than 50% of the sella turcica is occupied by CSF, and the pituitary gland thickness is less than or equal to 2 mm [10]. Figure 2 below shows the appearance of partial and complete ES on magnetic resonance imaging (MRI) of the brain. 

In some cases, patients may progress from having partial ES to complete ES. Carosi et al. revealed associated hormonal deteriorations in such patients when followed longitudinally for a median period of 58 months after being initially diagnosed with partial ES [10]. Proper diagnostic classification of patients with ES into these defined categories may help inform providers of potential etiologies of the condition and the likelihood of clinical progression and symptom development. 

Clinical presentation

By definition, ESS is the radiographic finding of ES combined with neurological, endocrine, and/or ophthalmological signs and symptoms. A detailed history and physical examination should be obtained.

Neurological

The most common neurological clinical presentation of ESS is headache [11]. Additional less common findings include cranial nerve palsies, dizziness, vertigo, syncope, seizures, back pain, and CSF rhinorrhea [11-13]. CSF rhinorrhea is a particularly high-risk finding as it indicates that the arachnoid diverticulum has eroded through the floor of the sella turcica, thus increasing the risk of retrograde meningitis. If this finding is present, referral to a neurosurgeon and/or otolaryngologist is advised [5,19]. 

Ophthalmological

Other than headache, the most common clinical presentation of ESS is visual disturbance [11]. In particular, visual field deficits, diplopia, blurred vision, and nystagmus have been reported. Due to the close anatomical relationship between the sella turcica and the optic chiasm, direct compression is thought to play a role in visual disturbances. Ophthalmologic examination is particularly important, as it can reveal signs of elevated ICP, such as papilledema. 

Endocrine

Endocrine presentation of ESS is highly variable, varying from subtle to life-threatening. The most common endocrinopathy is growth hormone deficiency, affecting up to 60% of patients. In adults, growth hormone deficiency can present with subtle findings of weight gain, central obesity, sarcopenia, weakness, depression, decreased bone mineral density, dyslipidemia, and insulin resistance. The second most common endocrinopathy is hyperprolactinemia, affecting up to 21% of patients. In ESS, the elevation is mild and rarely rises to clinical significance. Central hypogonadism affects up to 9% of patients with ESS, manifesting as fatigue, infertility, loss of libido, testicular atrophy, loss of body hair, gynecomastia, amenorrhea, and menstrual irregularity [7]. 

Other pituitary hormone deficiencies are less common in ESS and are reported in less than 7% of cases. Secondary adrenal insufficiency can result in a life-threatening adrenal crisis, presenting as shock and unresponsive to fluids or pressure. Arginine vasopressin deficiency has been reported in ESS, presenting as central diabetes insipidus with polyuria and polydipsia [14]. Central hypothyroidism presents with symptoms of decreased metabolic rate such as fatigue, cold intolerance, weight gain, bradycardia, constipation, or myxedema.

Diagnostic evaluation

ES can be diagnosed with either a computed tomography (CT) of the head or an MRI of the brain, the latter being the gold standard. Figure 2 in the “Classifications of empty sella” section shows MRI brain findings in partial and complete ES compared to a normal brain MRI. Currently, there is no standardized protocol for subsequent work-ups or management of the condition once it is diagnosed. Management for ES at present varies from some combination of hormonal evaluation, repeat imaging, and specialist referral to expectant management. 

Hormone evaluation is done by assessing pituitary function. Table 2 below summarizes the hormones typically included in a pituitary panel, with their associated reference ranges and functions. 

**Table 2:** Pituitary panel summary. IU: international units, L: liters, pg: picograms, mL: milliliters [29].

Hormone	Abbreviation	Reference range	Function			
Follicle-stimulating hormone	FSH	Men: 1.5-12.4 IU/L, women (follicular phase): 3.5-12.5 IU/L, women (luteal phase): 1.7-7.7 IU/L	Stimulates growth of ovarian follicles and spermatogenesis in men.			
Luteinizing hormone	LH	Men: 1.7-8.6 IU/L, women (follicular phase): 2.4-12.6 IU/L, women (luteal phase): 0.5-16.9 IU/L	Triggers ovulation and development of the corpus luteum in women; stimulates testosterone production in men.			
Adrenocorticotropic hormone	ACTH	9-52 pg/mL	Stimulates the adrenal glands to produce cortisol.			
Thyroid-stimulating hormone	TSH	0.4-4.0 mlU/L	Regulates the production of hormones by the thyroid gland.			
Growth hormone	GH	Men: <5 ng/mL, women: <10 ng/mL	Stimulates growth, cell reproduction, and regeneration.			
Prolactin	PRL	Men: 2-18 ng/mL, women (non-pregnant): 2-29 ng/mL, women (pregnant): 10-209 ng/mL	Promotes milk production in women after childbirth and has various other functions in both sexes.			

Management and treatment

The treatment of ES can be broadly categorized into conservative, medical, and surgical management modalities. Authors have varying recommendations with regard to the extent and combination of these treatment categories, with many suggesting some combination of the three. 

Conservative Management 

Conservative management, consisting of symptom monitoring and follow-up as appropriate, is recommended by authors such as Lundholm et al. They purport that no further investigations or treatments are required in patients with an isolated finding of ES. They state that ES is unlikely to ever require treatment, compared with ESS, which should be pharmacologically and/or surgically managed as appropriate [7].

Medical Management

Medical management is a commonly cited treatment recommendation, consisting of surveillance brain imaging, hormonal evaluation at the time of diagnosis, hormonal replacement therapy and endocrinology referral as necessary, and treatment of symptoms. Carosi et al. recommend repeat brain imaging at 12 months post-diagnosis to evaluate for any changes or progression [10]. Many authors recommend basic labs and hormonal evaluation, even in patients without any endocrine symptomatology, as there is a possibility of occult laboratory abnormalities prior to symptom development [10,11]. Carosi et al. recommend hormonal evaluation at the time of diagnosis, with follow-up between 24-36 months and correction of any imbalances in laboratory findings. Ucciferro et al. recommend hormonal corrections only in patients who become symptomatic, with a periodic re-evaluation of laboratory findings and endocrine symptoms at two to three-year intervals [6]. Various case reports cite specific interventions for the correction of hormonal imbalances, including levothyroxine, hydrocortisone, and cortisol [15,16]. In patients with discovered endocrine abnormalities, authors Miljic, Ekhzaimy, and Braiteh recommend patient referral to endocrinology [5,11,17]. Some patients with ES will later go on to develop ESS, and several authors suggest medical management tailored to patient-specific symptomatology [5-7,18]. Miljic et al. detail treatments such as osmotic diuretics or acetazolamide in patients with elevated ICP and weight loss in patients with obesity or obstructive sleep apnea [5]. In a case report by Al Mohareb et al., the authors recommended the use of the beta blocker Bisoprolol in a patient who was found to have both ES and a thyroid hormone receptor beta (THR-B) mutation causing hyperthyroid symptoms [18]. 

*Surgical Management* 

Another treatment modality is a surgical approach to ES. Indications for this more invasive management include various underlying etiologies that result in an elevated ICP. This may include both associations with PES, such as cortical hemorrhage, and etiologies of SES, such as space-occupying lesions or pituitary hemorrhage. Clinical findings indicating such underlying etiologies may require a consult to neurosurgery for potential surgical intervention. Miljic et al. recommend referral in patients with clinical evidence of elevated ICP, rhinorrhea, or associated visual disturbances [5]. 

As evidenced by the variation in suggested treatment modalities and follow-up recommendations, there is currently no standardized protocol for providers who encounter a patient with an incidental finding of ES. Current management relies on provider discretion, with the general consensus being management of any underlying causes of the condition, evaluation, and correction of hormonal imbalances as appropriate, symptom treatment, and follow-up with a general practitioner or specialist as necessary. 

Limitations

Given the limited availability of publications focusing specifically on the condition of ES, this literature review is founded on a relatively small sample size of publications. Much of the difficulty in writing this paper results from the vast abundance of literature on the topic of ESS but not on the incidental finding of ES. This is hypothesized because in cases of ES, where there is an absence of neurological, ophthalmological, or endocrine symptoms, adverse patient outcomes or substantial impact on quality of life are rare. The more benign nature of ES as compared to ESS attracts minimal excitement for publications despite its clinical prevalence.

## Conclusions

ES is a radiologic diagnosis of an enlarged sella turcica filled with CSF. This may present with some degree of pituitary gland and/or stalk compression. ESS is a diagnosis consisting of both this radiological finding and endocrine, neurological, and/or ophthalmological symptoms. While there are many publications on ESS, there is little research available on the topic of ES despite its moderate prevalence in the general population. ES typically presents in middle-aged patients, predominantly affecting females. While patients may not present with specific symptoms as they might in ESS, they are often incidentally diagnosed through brain imaging for non-specific clinical findings such as headaches. The gold standard for the diagnosis of ES is an MRI of the brain, although many patients first undergo a CT of the head. Management recommendations vary widely by publication, with some suggesting a very conservative approach and others advising serial follow-ups, repeat imaging, endocrine labs, or specialty referrals. The majority of patients diagnosed with ES have a good prognosis and will not develop debilitating or life-threatening symptoms. However, given the condition’s prevalence, potential critical underlying causes and associations, and possible clinical progression, providers should, at a minimum, be familiar with the condition and be comfortable managing it. This paper calls for a standardized protocol for providers encountering patients with ES to effectively rule out high-risk etiologies and monitor for progression to ESS. This will ensure optimized patient care and enable early management of any endocrine, neurological, and/or ophthalmological symptoms.

Based on the information gathered in the literature, we have synthesized the following clinical recommendations that may help inform management protocols for ES patients. Although patients are asymptomatic at the time of presentation, roughly half of patients with ES will have occult endocrine abnormalities; therefore, it is our recommendation that all patients with this radiographic finding undergo an endocrine panel. If the results of the panel reveal any abnormalities, we recommend patient referral to endocrinology. Additionally, patients with the diagnosis of ES and an unknown underlying etiology (PES) complaining of headaches and/or vision changes should be referred to ophthalmology for papilledema screening. Because certain causes of elevated ICP may not be evident on brain imaging, such as IIH or various vasculitic conditions, this screening will help eliminate undiagnosed etiologies. If papilledema is evident and no underlying cause such as hemorrhage, hydrocephalus, or a mass is identified, patients should be considered for more invasive procedures such as lumbar puncture with opening pressure or temporal artery biopsy based on clinical judgment. After this initial endocrine panel and ophthalmology referral, patients may be managed either conservatively, medically, or surgically with speciality referrals as appropriate. While these are our recommendations based on a thorough review of the literature, further studies are necessary to reach a consensus on the best clinical practices.
